# Evolutionary diversity in tropical tree communities peaks at intermediate precipitation

**DOI:** 10.1038/s41598-019-55621-w

**Published:** 2020-01-24

**Authors:** Danilo M. Neves, Kyle G. Dexter, Timothy R. Baker, Fernanda Coelho de Souza, Ary T. Oliveira-Filho, Luciano P. Queiroz, Haroldo C. Lima, Marcelo F. Simon, Gwilym P. Lewis, Ricardo A. Segovia, Luzmila Arroyo, Carlos Reynel, José L. Marcelo-Peña, Isau Huamantupa-Chuquimaco, Daniel Villarroel, G. Alexander Parada, Aniceto Daza, Reynaldo Linares-Palomino, Leandro V. Ferreira, Rafael P. Salomão, Geovane S. Siqueira, Marcelo T. Nascimento, Claudio N. Fraga, R. Toby Pennington

**Affiliations:** 1 Department of Botany, Federal University of Minas Gerais, Belo Horizonte, 31270-901 Brazil; 2 School of GeoSciences, University of Edinburgh, Edinburgh, EH9 3JN UK; 3 Royal Botanic Garden Edinburgh, Edinburgh, EH3 5LR UK; 4 School of Geography, University of Leeds, Leeds, LS2 9JT UK; 5 Departamento de Engenharia Florestal, Universidade de Brasília, Brasília, 70910-900 Brazil; 6 Departamento de Ciências Biológicas, Universidade Estadual de Feira de Santana, Feira de Santana, 44036-900 Brazil; 7 Instituto de Pesquisas Jardim Botânico do Rio de Janeiro, Rio de Janeiro, 22460-030 Brazil; 8 EMBRAPA Recursos Genéticos e Biotecnologia, Brasília, 70770-200 Brazil; 9 Comparative Plant and Fungal Biology Department, Royal Botanic Gardens, Kew, Richmond, Surrey, TW9 3AB UK; 10 Instituto de Ecología y Biodiversidad (IEB), Universidad de Chile, Santiago, Chile; 11 Museo de Historia Natural Noel Kempff Mercado, Universidad Autónoma Gabriel René Moreno, Santa Cruz de la Sierra, 2489 Bolivia; 12 Facultad de Ciencias Forestales, Universidad Nacional Agraria La Molina, Lima, 15024 Peru; 13 Universidad Nacional de San Antonio Abad del Cusco, Cusco, 08000 Peru; 14 Smithsonian Conservation Biology Institute, Lima, 15001 Peru; 15 Coordenação de Botânica, Museu Paraense Emilio Goeldi, Belém, 66077-530 Brazil; 16 Universidade Federal Rural da Amazônia, Belém, 66077-530 Brazil; 17 Reserva Natural Vale, Linhares, 29909-030 Brazil; 18 Laboratório de Ciências Ambientais, Centro de Biociências e Biotecnologia, Universidade Estadual do Norte Fluminense, Campos dos Goytacazes, 28013-620 Brazil; 19 Department of Geography, University of Exeter, Exeter, EX4 4RJ UK

**Keywords:** Ecology, Evolution

## Abstract

Global patterns of species and evolutionary diversity in plants are primarily determined by a temperature gradient, but precipitation gradients may be more important within the tropics, where plant species richness is positively associated with the amount of rainfall. The impact of precipitation on the distribution of evolutionary diversity, however, is largely unexplored. Here we detail how evolutionary diversity varies along precipitation gradients by bringing together a comprehensive database on the composition of angiosperm tree communities across lowland tropical South America (2,025 inventories from wet to arid biomes), and a new, large-scale phylogenetic hypothesis for the genera that occur in these ecosystems. We find a marked reduction in the evolutionary diversity of communities at low precipitation. However, unlike species richness, evolutionary diversity does not continually increase with rainfall. Rather, our results show that the greatest evolutionary diversity is found in intermediate precipitation regimes, and that there is a decline in evolutionary diversity above 1,490 mm of mean annual rainfall. If conservation is to prioritise evolutionary diversity, areas of intermediate precipitation that are found in the South American ‘arc of deforestation’, but which have been neglected in the design of protected area networks in the tropics, merit increased conservation attention.

## Introduction

Given predictions of increased temperature and precipitation extremes^1^, it is imperative to understand the mechanisms driving the distribution of biodiversity along climatic gradients. Recent macroecological studies^2,3^ have shown that the inability of most plant lineages to survive regular frost may underlie the latitudinal diversity gradient for flowering plants (angiosperms), which are most species-rich and evolutionarily diverse in the tropics^3–9^. While there is compelling evidence for the importance of frost in shaping patterns of species and evolutionary diversity of plants^2,3^, drought is another major axis of environmental stress that merits attention. Patterns of variation in angiosperm species richness across tropical drought gradients are clear: species richness is highest under the wettest conditions^4–8^. However, unlike frost, there are no studies that examine the role of drought in driving patterns of evolutionary diversity at large spatial scales.

Our understanding of the influence of evolutionary history on major gradients in the global distribution of biodiversity is framed by two hypotheses. The importance of climatic history for the latitudinal diversity gradient has given rise to the ‘Out of the Tropics’ Hypothesis^10^ (OTH), which builds upon the assumption that many major clades of animals and plants originated and initially diversified when the Earth’s climate was primarily warm and wet^10–13^. The Tropical Conservatism Hypothesis^11,12,14,15^ (TCH) is complementary to the OTH, and proposes that lineages associated with climatic extremes (e.g., strong seasonal frost and drought) are descendants of clades from warmer and wetter regions and derived from a small subset of lineages that developed the necessary innovations to thrive in harsh conditions. Thus, if greater time for diversification in the wet tropics and ancestral preferences for such conditions were the primary forces shaping diversity patterns across large-scale gradients of both temperature^2,3^ and precipitation, we would expect wet tropical environments to hold the greatest amount of evolutionary diversity.

Alternatively, phylogenetic conservatism for harsh environments may play a distinct role in shaping evolutionary diversity patterns across climatic gradients. Phylogenetic conservatism for dry biomes has been demonstrated for multiple plant clades, and these can be tens of millions of years old^15^. If these clades spill out of dry extremes into areas with intermediate precipitation to coexist with members of the majority of angiosperm clades that prefer high rainfall environments, we may expect areas with intermediate precipitation to have higher amounts of evolutionary diversity because they can contain specialised lineages from both extremes. We refer to this alternative hypothesis as the ‘Environmental Crossroads Hypothesis’ (ECH). Previous research on patterns of species richness across climatic gradients have both supported^16–18^ and failed to support the ECH^4–8^, but we do not know of studies that have tested it from an evolutionary perspective.

**Figure 1:**
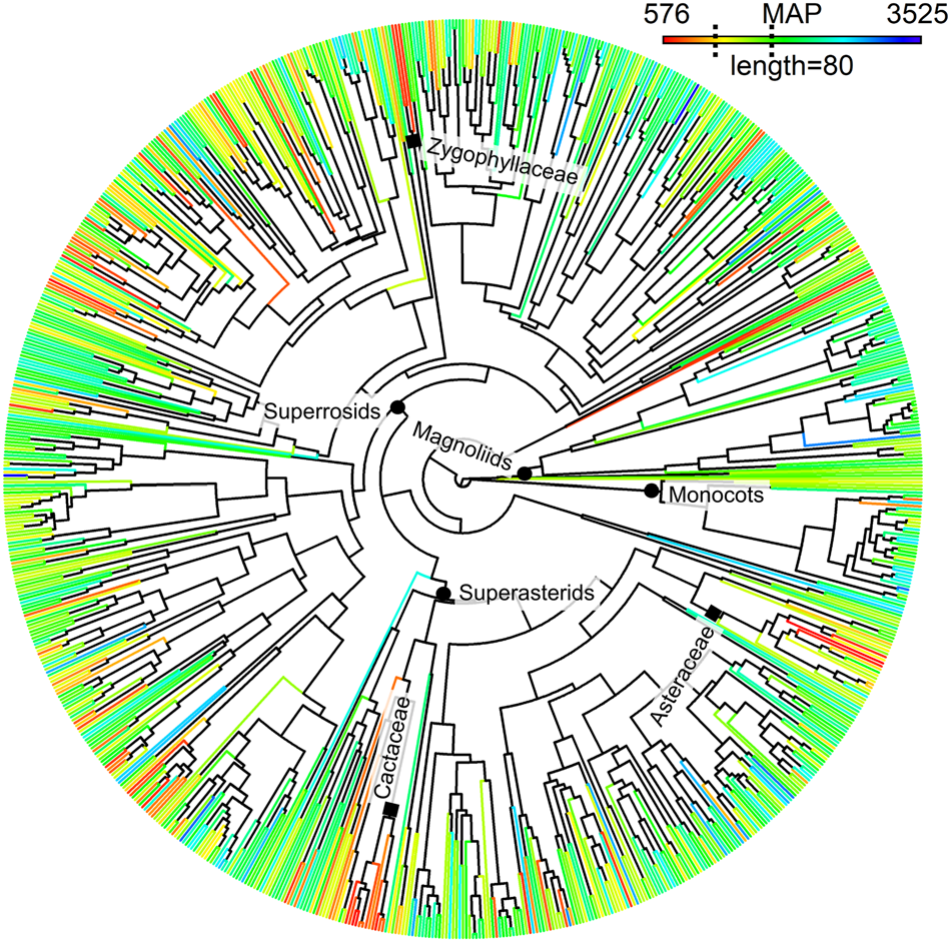
Time-calibrated molecular phylogeny of 852 angiosperm genera found in lowland tree communities of tropical South America. Phylogenetic reconstruction based on sequences of rbcL and matK plastid regions from plants collected during fieldwork or available in GenBank. Tree topology and divergence times of taxa were estimated using a Bayesian Markov Chain Monte Carlo approach. Branch lengths were time-scaled using a relaxed molecular clock with fossil-based age constraints implemented on nodes (Appendix 1). Colours represent mean annual precipitation (MAP), with warmer colours indicating drier conditions. The minimum and maximum MAP are given. Scale (length) is in myrs and is equivalent to branch lengths in the phylogeny (80 myrs). Dotted lines indicate 1,200 mm and 1,800 mm of MAP. Black circles indicate the nodes comprising lineages from the major angiosperm clades: Magnoliids, Monocots, Superrosids, Superasterids. Black squares indicate nodes comprising some of the dry-adapted lineages that are absent or have a much lower frequency of occurrence in wet environments, at least as trees (e.g., Cactaceae, Zygophyllaceae, Asteraceae; see Discussion).

Testing the validity of these hypotheses in an evolutionary context requires large-scale data on the distribution and phylogenetic relationships of multiple lineages along gradients of environmental stress. In contrast to prior studies of evolutionary diversity in the tropics^19,20^, this study is based on continental-scale sampling that sufficiently covers tropical climatic gradients. We use a database of 2,025 tree communities from moist forests to savannas and dry woodlands, covering the full breadth of environmental space of lowland tropical South America (http://neotroptree.info). We then combine it with a new time-scaled molecular phylogeny for 852 angiosperm genera (Figs. 1 and S1), which represent 93% of the genera occurrences and 99% of species occurrences in the database.

Quantifying evolutionary diversity is of interest from both biodiversity conservation and ecosystem function perspectives. The importance of conserving areas of high evolutionary diversity has been widely recognised^21,22^, and recent studies show that ecosystem function should also be higher in areas of greater evolutionary diversity^23–28^ (*e.g*., higher plant community productivity^25–28^). While many metrics have been developed to quantify evolutionary diversity in ecological communities^29^, we employ Faith’s Phylogenetic Diversity^21^ (the sum of all branch lengths in a given community; PD), as it aligns most closely with the richness dimension of evolutionary diversity^29^. However, raw PD is strongly correlated with taxonomic richness^20,22^, thus being strongly affected by sample effort. Our dataset includes a large number of sites across the breadth of environmental gradients, but the sampling effort varies, which will influence the number of taxa found, and raw PD values. We therefore focus our analyses on the standardised effect size of PD, a metric we refer to as lineage diversity – a measure of the excess or deficit of PD given the number of genera found in a sample. Specifically, we use lineage diversity estimates to address the predictions stemming from the OTH, TCH and ECH, and to provide conservation insights that go beyond approaches relying upon species richness alone.

## Results

**Figure 2:**
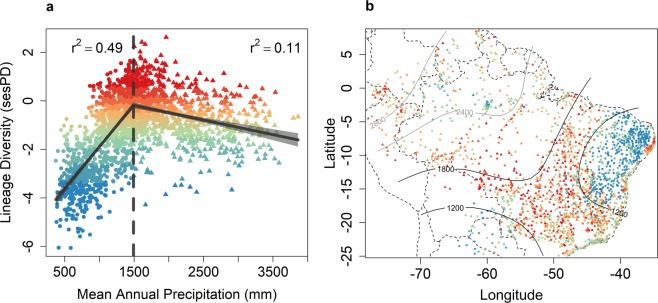
Relationship between mean annual precipitation (MAP) and lineage diversity (standardised effect size of phylogenetic diversity, a measure of the evolutionary diversity of communities) across 2,025 lowland tree communities of tropical South America. (**a**) Effect of MAP on lineage diversity (LD). Break point (1,490 mm) was determined by piecewise regression. r^2^ = coefficient of determination from generalized least squares (GLS) models that account for spatial autocorrelation. GLS was calculated for before (y = 0.002326x − 2.634307) and after (y = −0.0006x + 0.711) the break point. (**b**) Geographical variation of lineage diversity and MAP. Colours of the symbols illustrate lineage diversity and are identical to colours in (**a**) (warmer colours indicate higher values). Circles indicate communities below and triangles above the precipitation break point (1,490 mm). Grey areas around the curves in (**a**) are 99% confidence intervals. These represent, for a given value of MAP, the interval estimate for the mean of LD, thus reflecting the uncertainty around this mean. Dashed lines represent national borders and contours represent mean annual precipitation in (**b**).

We found strong and clear phylogenetic signal for the precipitation conditions in which genera occur (λ = 0.5; P < 0.001). Closely related genera are more likely to occur under a similar precipitation regime (measured as mean annual precipitation; henceforth MAP). In addition, the clades that are comprised largely of genera in dry regions (MAP <1200 mm, red and orange in Fig. 1) tend to be young compared to clades comprised largely of genera in wetter regions (MAP >1800 mm, green and blue in Fig. 1). These results are expected and in agreement with the OTC, TCH and ECH. Contrary to predictions from the OTH or TCH, whereby highest lineage diversity would be found in wetter regions, we find that communities in areas with intermediate MAP have the highest lineage diversity (Fig. 2). A piecewise regression model, whereby a break-point is identified between two non-overlapping linear regressions, provided a better fit to the lineage diversity and MAP relationship (r^2^ = 0.48; *P < *0.001) than a linear regression (r^2^ = 0.16; *P < *0.001) or a quadratic polynomial, ‘hump-shaped’ model (r^2^ = 0.42; *P < *0.001) (Table S1 and Fig. S2 in Supplementary Materials).

The break-point that represents the peak in lineage diversity was identified at 1,490 mm of MAP, with lineage diversity declining as MAP increases or decreases from this value (Fig. 2a). Below the threshold (in drier conditions), MAP explained 49% of the observed deviance in a generalized least squares framework (GLS) that accounts for spatial autocorrelation (Fig. 2a). Above the threshold, MAP explained 11% of the deviance (Fig. 2a).

**Figure 3:**
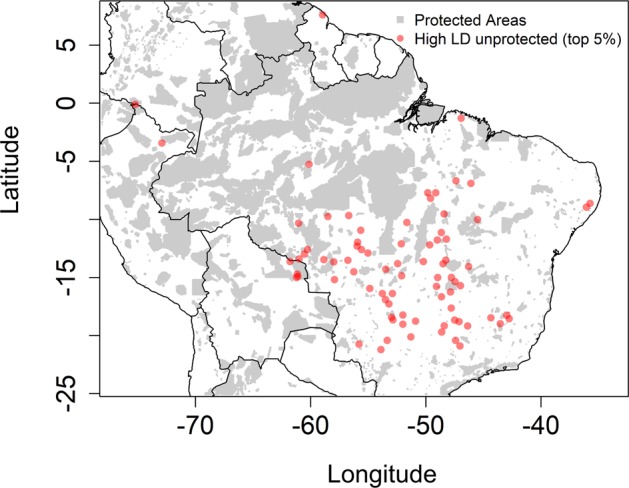
Conservation assessment of lineage diversity across lowland tree communities in tropical South America. Distribution of the top 5% unprotected tree communities with highest lineage diversity (80 sites; red circles). Unprotected status was determined by overlaying the distribution of our sites on to the coverage of protected areas across South America.

Assessing the distribution of communities that are not covered by the existing network of protected areas in South America, we find that the top 5% communities (80 unprotected sites) with highest lineage diversity are largely found across the intermediate MAP region (Fig. 3, Appendix 2). In addition, these sites are found in municipalities that have lost 66,685 Km^2^ of their natural cover over the last 30 years (more than twice the size of Belgium; Appendix 2).

When considering alternative measures of climatic water availability, such as climatic water deficit, precipitation seasonality and water deficit duration, we also found a peak in lineage diversity at intermediate values (Fig. S3). In contrast, we found no relationship between temperature-related variables and lineage diversity (Fig. S4). These results are robust to (*i*) including unsampled genera in the phylogeny^30^ (Fig. S5), (*ii*) controlling for potential richness-dependence of our lineage diversity metric^31^ (Fig. S6), (*iii*) different temporal calibration methods (Fig. S7), and are consistent (*iv*) across a set of phylogenies from the posterior distribution (Fig. S8) and (*v*) when using a species-level phylogeny (Fig. S9).

## Discussion

The mismatch that we uncovered between higher species richness in wetter environments^4–7^ and higher lineage diversity in areas under intermediate MAP (Fig. 2a,b) indicates that the distribution of evolutionary diversity in neotropical tree communities might not be as simplistic as previously thought, based on studies with limited coverage of environmental gradients^19,20,32^. Our results support the ECH by showing that communities at the threshold between wet and dry environments (1,200 mm ≤ MAP < 1,800 mm; Fig. 2b) contain both wet and dry-adapted lineages and therefore high evolutionary diversity. The dramatically reduced lineage diversity across communities at the dry extreme (MAP < 1,200 mm; i.e., semi-arid woodlands; blue circles in Fig. 2b) may reflect the limited ability of wet-adapted lineages to survive in dry climates, with lineages there representing a phylogenetically nested subset of the continental pool that can tolerate low MAP.

Towards the wet extreme (MAP ≥ 1,800 mm), the unexpectedly reduced lineage diversity suggests that intermediate MAP may also be a threshold for the dry-adapted subset of lineages. These results are in agreement with phylogenetic studies showing that plant lineages in seasonally dry environments (e.g., *caatinga* woodlands; blue circles in northeastern Brazil in Fig. 2b) are often confined to these environments over evolutionary timescales^15^. Such evidence for phylogenetic conservatism in dry environments, combined with our results showing highest lineage diversity in intermediate MAP, bring support to the ECH, whereby highest evolutionary diversity along any environmental gradient, if sampled extensively as in this study, will be found in intermediate conditions because communities located at one environmental extreme (e.g. hyper-wet) are likely to be missing lineages adapted and confined to the other extreme (e.g., highly seasonally dry; Fig. 1). Meanwhile, the high species richness in wet areas, despite having lower lineage diversity, may be due to recent species diversification in wet areas. The wet tropics of South America have been a cradle of recent lineage diversification, at least for some clades^33–35^ (although see Fine *et al*.^36^ for a counterexample). Nonetheless, well-resolved species-level phylogenies are needed to assess the ubiquity of high recent diversification rates in the wet tropics and the role that variable diversification rates may play in the observed patterns.

Our results are of relevance for conservation strategies in South America that take into account evolutionary diversity^37^. One approach to conserving maximum tropical plant lineage diversity would be to protect different communities at either end of the precipitation gradient, and our study therefore highlights the unique and often over-looked lineages found in dry communities (Fig. 1 and Appendix 2) that are currently under-protected^38^. Our results also highlight an additional approach, which would be to protect the evolutionarily diverse communities found at intermediate precipitation. The intermediate MAP region includes ecosystems within the ‘arc of deforestation’ where habitat alteration has been rapid and pervasive^39^, and where climate change effects may be severe^40^. Nonetheless, these evolutionarily diverse communities are largely unprotected by the existing network of protected areas in South America (Fig. 3; see Appendix 2 for detailed information on these communities).

Per unit area, protection of tree communities in the arc of deforestation and in Central Brazil may conserve the widest range of evolutionary lineages of South American trees. Because the amount of evolutionary diversity in communities is associated with resilience to climate change^41^, ensuring that these communities are conserved, in conjunction with wetter and drier habitats, is important to ensure that the full evolutionary diversity of neotropical forests is preserved in the face of land-use and climate change.

## Methods

### Datasets

We extracted climatic data and inventories of 2,025 tree communities from NeoTropTree (NTT; http://neotroptree.info; Appendix 2). Taxonomic nomenclature was made consistent by querying species names against Tropicos (http://tropicos.org) and Flora do Brasil (http://floradobrasil.jbrj.gov.br). The NTT database includes environmental variables for all its sites, derived from multiple sources (see Supplementary Methods for details). We constructed a molecular phylogeny for 1,100 lowland tropical tree genera from South America by sequencing the *rbcL* and *matK* plastid regions from plants collected during fieldwork or from GenBank (http://www.ncbi.nlm.nih.gov/) (Appendix 3). We aligned the genetic data using MAFFT (http://align.bmr.kyushu-u.ac.jp/mafft), and performed Bayesian phylogenetic inference using BEAST v.1.8.2 in the CIPRES Science Gateway (https://www.phylo.org; see Supplementary Methods for further details). Branch lengths were time-scaled using a relaxed molecular clock with fossil-based age constraints implemented on 86 nodes (Appendix 1). In order to assess the robustness of results to temporal calibration approach (Fig. S7), we also generated a maximum likelihood phylogeny using RAxML v8^42^, and time-scaled it using penalized likelihood and the same fossil calibrations^43^, via the TreePL software^44^ (available at https://github.com/blackrim/treePL). These phylogenies were then pruned to the 852 genera in the community matrix for downstream analyses.

### Data analyses

We determined the precipitation niche of each genus by calculating the mean MAP for sites at which it occurred. We then color-coded these values at terminal branches in order to visualise their phylogenetic distribution. We estimated phylogenetic signal for mean MAP by using Pagel’s *lambda*^45^, which varies from 0 to 1. A value of 1 indicates a strong relationship between phylogenetic position of genera and their mean MAP, while a value of 0 indicates that there is no relationship between mean MAP and the phylogeny. We assessed the significance of *lambda* using a likelihood ratio test. We conducted the trait mapping and phylogenetic signal analyses using the phyloch^46^ and phytools^47^ packages in the R Statistical Environment^48^.

We calculated lineage diversity as the total phylogenetic branch length (PD) in communities^21^ standardized for genus-level richness (i.e., standardized effect size of PD; sesPD^49^), a metric we refer to as lineage diversity. This metric measures how PD deviates from a null expectation, generated by randomly shuffling the tips of the phylogeny and recalculating PD in communities^49^. We tested whether the lineage diversity (LD) results are robust to including missing taxa by randomly inserting into the phylogeny the 68 genera that are present in the genus-by-site matrix but lacking appropriate molecular data. This consisted of determining the most derived consensus clade for each missing taxon (MDCC; i.e., family, subfamily, tribe or subtribe^30,50,51^), and then inserting them in random positions within their MDCCs. We repeated this procedure 100 times for each of 100 trees sampled from across the posterior distribution (see Fig. S5 and ‘Phylogenetic tree’ in Supplementary Methods).

We tested whether the decreasing LD in wet communities (mean annual precipitation ≥1,800 mm) is a richness-dependent artefact^31^ by calculating LD using a set of 100 genus-by-site matrices randomly rarefacted to 86 genera (1/4 of maximum generic richness^31^ in wet communities) and phylogenetic trees pruned to the genus pool in each matrix. Because communities found in drier conditions (mean annual precipitation <1,800 mm) show a pattern of decreasing lineage diversity towards drier, species-poor environments and richness-dependent artefacts would operate in the opposite direction^31^, we tested for a potential richness-dependence using wet communities only. These analyses generated 100 LD values for each of the 519 wet communities, which were used to calculate mean values per community (Fig. S6).

We tested whether our results are robust to phylogenetic uncertainty by calculating LD across a set of 100 phylogenies from the posterior distribution (see Fig. S8 and ‘Phylogenetic Tree’ in Supplementary Methods). Finally, we calculated LD using a simulated species-level phylogeny (see Fig. S9 and ‘Phylogenetic tree’ in Supplementary Methods).

We assessed the goodness-of-fit between lineage diversity and climatic variables (see ‘Database’ in Supplementary Methods) through adjusted coefficients of determination, AIC values and significance tests for linear, quadratic and piecewise regressions^52^ (Table S1). We assessed variation in the adjusted coefficients of determination for these regressions using the full genus-by-site matrix (920 genera) and a set of 10,000 phylogenetic trees that include the 68 missing genera (see imputation methods above and Fig. S2).

Because spatial autocorrelation can inflate type I error in traditional statistical tests and affect parameter estimates, we accounted for it by performing generalized least squares analyses with four different spatial structures: exponential, Gaussian, linear and spherical. Model selection was based on the minimization of AIC values; i.e., ∆AIC relative to the null model without spatial autocorrelation. An exponential spatial structure accounted best for spatial autocorrelation relative to other spatial structures in both models; i.e., for before (∆AIC = −456.4) and after (∆AIC = −208.9) the break-point in lineage diversity (Fig. 2a). We conducted the spatial analyses using the nlme package^53^ in R^48^.

### Conservation assessment

We assessed the protection status (protected or unprotected) of our 2,025 tree communities by overlaying their distribution on to the coverage of protected areas across South America (Appendix 2). We used conservation units from the World Database of Protected Areas (IUCN & UNEP - WCMC, www.protectedplanet.net; downloaded on July 2019). We also computed the loss of natural cover over the last 30 years for the municipalities where our tree communities are found (c.90% of our sites; Appendix 2) using information from the MapBiomas Project (http://mapbiomas.org).

## Supplementary information

Supplementary Information

Appendix 1

Appendix 2

Appendix 3

## Data Availability

Time-calibrated molecular phylogenies are deposited at the Dryad Digital Repository (10.5061/dryad.gf1vhhmk0). A full description with details of data accessibility for Neo-TropTree can be found at http://www.neotroptree.info/.
