# Supplementary material for: Evolutionary diversity in tropical tree communities peaks at intermediate precipitation
Source: Sci Rep. 2020 Jan 24;10:1188. doi: 10.1038/s41598-019-55621-w (PMC6981197; doi:10.1038/s41598-019-55621-w)
Supplement: Supplementary file 1 — Supplementary Information [file 41598_2019_55621_MOESM1_ESM.docx]

Supplementary Materials for

**Evolutionary diversity in tropical tree communities peaks at intermediate precipitation**

Danilo M. Neves, Kyle G. Dexter, Timothy R. Baker, Fernanda Coelho de Souza, Ary T. Oliveira-Filho_,_ Luciano P. Queiroz, Haroldo C. Lima, Marcelo F. Simon, Gwilym P. Lewis, Ricardo A. Segovia, Luzmila Arroyo, Carlos Reynel, José L. Marcelo-Peña, Isau Huamantupa-Chuquimaco, Daniel Villarroel, G. Alexander Parada, Aniceto Daza, Reynaldo Linares-Palomino, Leandro V. Ferreira, Rafael P. Salomão, Geovane S. Siqueira, Marcelo T. Nascimento, Claudio N. Fraga, R. Toby Pennington

Correspondence to: dneves@icb.ufmg.br

**This file includes:**

Materials and Methods (page 3)

Table S1 (page 9)

Figures S1-S9 (page 10)

Supplementary References 54-81 (page 25)

**Other Supplementary material for this manuscript includes the following:**

**Appendix 1.** List of angiosperm clades (nodes) used to date the phylogeny via fossil-derived calibrations, taxa assigned as a reference for fossil placement, minimum age used as offset, mean age of the clades, and their respective sources. Ages in myrs.

**Appendix 2.** List of 2,025 tree community surveys used in this study with their respective metadata, including sources, genus and species richness, lineage diversity (standardised effect size of phylogenetic diversity), climatic variables, protection status, and loss of native area over the last 30 years in the municipality where these communities occur.

**Appendix 3.** List of 1,100 angiosperm genera used in the phylogenetic reconstructions, their respective sources of *rbcL* and *matK* sequences, GenBank accession numbers, when applicable; collectors, collector numbers and herbaria where vouchers were deposited, when applicable. RBG = Royal Botanic Garden Edinburgh. Asterisk indicates the 835 genera used in the community phylogenetic analyses.

**Materials and Methods**

Database

We extracted the floristics dataset from the NeoTropTree (NTT) database (http://neotroptree.info), which consists of checklists of tree species compiled for geo-referenced sites, extending from southern Florida (U.S.A.) and Mexico to Patagonia. Trees here are defined as woody and succulent, freestanding plant species (i.e., lianas excluded), with branching above 1m. NTT currently holds 5,954 sites/checklists, 17,240 tree species and 1,014,656 occurrence records. A site/checklist in NTT is defined by a single vegetation type, following the classification system proposed by Oliveira-Filho^54^, contained in a circular area with a 10 km diameter. Where two or more vegetation types co-occur in one 10-km area, there may be two geographically overlapping sites in the NTT database, each for a distinct vegetation type.

The data were originally compiled from an extensive survey of published and unpublished literature (e.g., PhD theses, environmental consultancy reports), particularly those comprising floristic surveys and forest inventories. Moreover, new species occurrence records obtained from major herbaria and taxonomic monographs have been added to the checklists when they come from within a 5 km radius from the center of the original NTT site, and within the same vegetation type (see Appendix 2 for a list of data sources). All species and their occurrence records were checked regarding current taxonomic and geographical circumscriptions, as defined by the team of specialists responsible for the online projects *Flora do Brasil* (available at http://floradobrasil.jbrj.gov.br/) and *Tropicos* (available at http://tropicos.org). The compilation of NTT avoided, therefore, the inclusion of occurrence records with doubtful identification, location or vegetation type, and sites with an indication of high anthropogenic disturbance. We also excluded checklists with very low species richness (< 20 species) because, within the tropics, this is often due to low sampling/collecting efforts and results in poor descriptive power. While we acknowledge the probable existence of identification errors in the aforementioned studies, we believe that our main results in this study are robust to these misidentifications because (i) most misidentifications are likely to be among species within the same genus, and (ii) there would not be any systematic variation of genus misidentification rate along environmental gradients that would introduce bias into our analyses.

We restricted analyses to the tropical lowlands of South America, thus excluding frost-affected sites as well as NTT sites above 1,000m elevation or below 23^o^ S latitude. The dataset extracted from NTT consisted of 2,025 tree communities (Appendix 2). Lowland forests, savannas and semi-arid woodlands originally covered an area of *ca.* 11.5 million km^2^, corresponding to 65% of the land surface of South America^55-57^. Rain forests represent the largest areas (*ca.* 7.3 million km^2^) and house the higher proportion of South American plant diversity^58,659^. They occur primarily in the Amazon basin and along the Atlantic coast of Brazil and are separated by the so-called “dry diagonal”, a corridor that includes the Caatinga (semi-arid thorn woodlands) of northeastern Brazil, the Cerrado (woody savannas) of central Brazil, and the Chaco (semi-arid thorn woodlands) of Paraguay, Argentina and Bolivia^60^. The Cerrado, besides the predominant savanna vegetation, also comprises scattered patches of seasonally dry forests on fertile soils^61-64^ and riverine forests along waterlogged riverbeds^63^, although these two formations show higher floristic affinities with semi-arid thorn woodlands of Caatinga and Atlantic rain forests^65^, respectively. The full genus-by-site matrix consisted of 498 Amazonian rain forests, 397 Atlantic rain forests, 389 *caatinga* thorn woodlands, 37 *chaco* thorn woodlands, 310 *cerrado* woody savannas, 109 seasonally dry forests and 291 riverine forests, with presence/absence data for 920 tree genera and a total of 252,764 presences.

The NTT database also includes environmental variables for all its sites, derived from multiple sources (at a 30 arc-second resolution; Appendix 2). Mean annual precipitation, precipitation seasonality, mean annual temperature and mean minimum temperature were obtained from WorldClim 1.4 data layers^66^. WorldClim monthly temperatures and precipitation were also interpolated to obtain values for 5-day intervals by applying sinusoidal functions centered at day 15 of each month. These functions yielded values for days 1, 5, 10, 20, 25 and 30, which were used to generate a water deficit duration (days) variable from Walter’s Climate Diagrams^67^. Climatic Water Deficit was extracted from Chave et al.^68^, and is an estimate in millimeters per year of the cumulative difference between precipitation and potential evapotranspiration over the consecutive months within a year when evapotranspiration is higher than precipitation (i.e. over the dry season).

Phylogenetic tree

We constructed a genus-level phylogeny comprising 1,100 angiosperm genera found in lowland tropical South America, following protocols developed by Dexter & Chave^69^. We used two chloroplast DNA gene regions: *rbcL* and *matK*. These genes were chosen based on their universality, data availability, typical sequence quality, degree of genus-level discrimination, sequencing costs and because they are recommended for standard DNA barcoding in plants^70-72^. We generated 198 novel *rbcL* and 264 novel *matK* sequences from leaf fragments collected during extensive fieldwork across South America (all sequences generated in this study have an associated voucher; see Appendix 3 for a list of accession numbers and voucher information). Further sequences were also obtained through Genbank (http://www.ncbi.nlm.nih.gov/), and restricted to accessions that had an associated voucher. We had both *rbcL* and *matK* sequences for 808 genera (73%), only *rbcL* for 128 genera (12%) and only *matK* for 163 genera (15%). Sequences that were unavailable for a single region for a given genus were left as missing data. Exploratory sequence alignments and phylogenetic reconstructions enabled us to exclude sequences that were likely to represent taxonomic misidentifications. The details of DNA extraction, PCR, and DNA sequencing protocols can be found in Gonzales *et al*.^73^ A list of sampled genera, their respective family and GenBank accession numbers are available in Appendix 3.

We conducted multiple sequence alignments, separately for each region, using MAFFT v.6.822^74^, followed by manual adjustments in Mesquite (http://mesquiteproject.org). After manual alignments, we reduced remaining alignment issues by removing all sites which were missing data for >99% of genera. All *rbcL* and *matK* sequences were then combined to generate a starting maximum likelihood tree using RAxML v.7.2.7 in the CIPRES Science Gateway (https://www.phylo.org). A topological constraint specifying the major relationships among angiosperm orders was imposed based on the Angiosperm Phylogeny Group^75^. The early-branching angiosperm *Nymphaea alba* L. (Nymphaeaceae) was specified as an outgroup. This initial phylogeny was made ultrametric by using nonparametric rate smoothing method^43^ implemented in the ape package^76^ in the R statistical environment^48^, and then used as a starting tree in a Bayesian Markov Chain Monte Carlo (MCMC) approach to simultaneously estimate tree topology and divergence times of taxa^77^. These analyses were performed by using BEAST v.1.8.2 on the CIPRES server. An uncorrelated lognormal relaxed molecular clock was implemented, and the tree prior was a Birth-Death Incomplete Sampling model of speciation^78^. We used 86 previously compiled fossil-based age constraints to calibrate node ages^79,80^ (see Appendix 1 for a list of priors and their respective nodes). Internal nodes were constrained using a log-normal distribution with a mean value equal to the fossil age, a standard deviation of 2 and a hard constraint for a minimum age equal to 80% of the estimated fossil age. No constraints were placed on the root age of the tree. We optimized operator settings before conducting the final runs by using a preliminary tree in test runs of 10^6^ generations.

We carried out three independent MCMC runs for 70.2 x 10^5^, 80.3 x 10^5^ and 58.6 x 10^5^ generations, under the same estimation conditions. We excluded burn-ins of 10^3^ and 2 x 10^3^ generations for the first two and third runs, respectively. We used LogCombiner to combine the three independent runs before sampling 282 trees evenly spaced across the posterior distribution, which were used to assemble a consensus tree. The consensus tree was assembled following the all compatible consensus rule; i.e., we choose the topology of a given node based on the relationship found in a plurality of trees from across the posterior distribution. This ensures a fully bifurcating topology that represents the most probable relationships of taxa. Lastly, we used TreeAnnotator (http://beast.bio.ed.ac.uk/treeannotator) to assign branch-lengths and divergence times (node heights) as the mean values from across the posterior distribution.

We also generated a species-level phylogeny, using the genus-level phylogeny as a basis. This consisted of imputing all 8,174 species in the floristics dataset by simulating a random birth-death phylogeny for each genus, using a speciation rate of 1 and an extinction rate of 0.9. We conducted these simulations in the TreeSim^81^ package in R^48^. This procedure produced a fully bifurcating phylogeny for each genus with the number of tips (i.e. species) corresponding to the number of species in the genus in our dataset. Importantly, we retained the stem age of the genus as estimated using our temporally calibrated phylogeny, while the crown age of genera was a product of the simulation (the mean expectation under a coalescent process is an age half that of the crown age, but there is variability around this expectation). The result of our approach is that the phylogenetic diversity represented by an individual species is proportional to the stem age of the genus divided by the species richness of the genus.

|  | Linear | | | Quadratic | | | Piecewise | | |
| --- | --- | --- | --- | --- | --- | --- | --- | --- | --- |
|  | Adjusted r^2^ | P-value | AIC | Adjusted r^2^ | P-value | AIC | Adjusted r^2^ | P-value | AIC |
| Mean Annual Precipitation | 0.1584 | < 2.2e-16 | 6745.72 | 0.4234 | < 2.2e-16 | 5981.21 | 0.4793 | < 2.2e-16 | 5775.42 |
| Climatic Water Deficit | 0.2562 | < 2.2e-16 | 6495.81 | 0.3626 | < 2.2e-16 | 6184.11 | 0.4176 | < 2.2e-16 | 6002.40 |
| Precipitation Seasonality | 0.0207 | 4.70e-11 | 7052.70 | 0.1127 | < 2.2e-16 | 6853.84 | 0.2068 | 4.76e-15 | 6627.81 |
| Water Deficit Duration | 0.1188 | < 2.2e-16 | 6838.86 | 0.3665 | < 2.2e-16 | 6171.63 | 0.4121 | < 2.2e-16 | 6021.34 |
| Mean Annual Temperature | -0.0003 | 0.5094 | 7095.61 | 0.0092 | 3.33e-05 | 7077.40 | 0.0170 | 1.40e-05 | 7062.45 |
| Mean Minimum Temperature | 0.0120 | 4.60e-07 | 7070.59 | 0.0243 | 6.02e-12 | 7046.30 | 0.0347 | 2.73e-11 | 7025.53 |

**Table S1 |** Goodness-of-fit-between lineage diversity (standardised effect size of phylogenetic diversity, a metric of evolutionary diversity in communities) and climatic variables across 2,025 lowland tree communities of tropical South America. Adjusted r^2^ = adjusted coefficient of determination; AIC = Akaike Information Criterion.


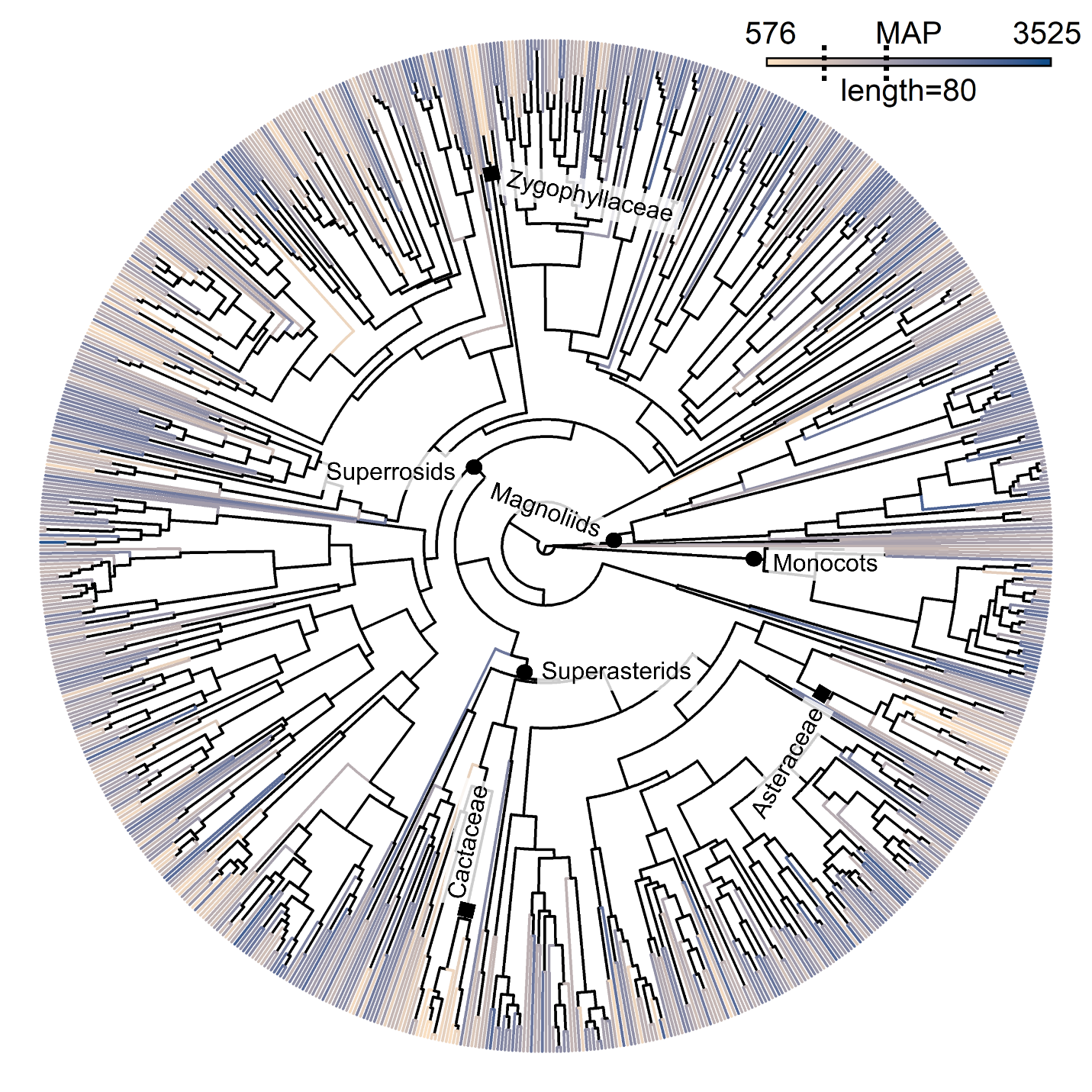


**Figure S1 | Time-calibrated molecular phylogeny of 852 angiosperm genera found in lowland tree communities of tropical South America.** The same as Fig. 1 but with colour-blind-friendly colour coding. Phylogenetic reconstruction based on sequences of rbcL and matK plastid regions from plants collected during fieldwork or available in GenBank. Tree topology and divergence times of taxa were estimated using a Bayesian Markov Chain Monte Carlo approach. Branch lengths were time-scaled using a relaxed molecular clock with fossil-based age constraints implemented on nodes (Appendix 1). Colours represent mean annual precipitation (MAP), with warmer colours indicating drier conditions. The minimum and maximum MAP are given. Scale (length) is in myrs and is equivalent to branch lengths in the phylogeny (80 myrs). Dotted lines indicate 1,200mm and 1,800mm of MAP. Black circles indicate the nodes comprising lineages from the major angiosperm clades: Magnoliids, Monocots, Superrosids, Superasterids. Black squares indicate nodes comprising some of the dry-adapted lineages that are absent or have a much lower frequency of occurrence in wet environments (e.g., Cactaceae, Zygophyllaceae, Asteraceae; see Discussion).


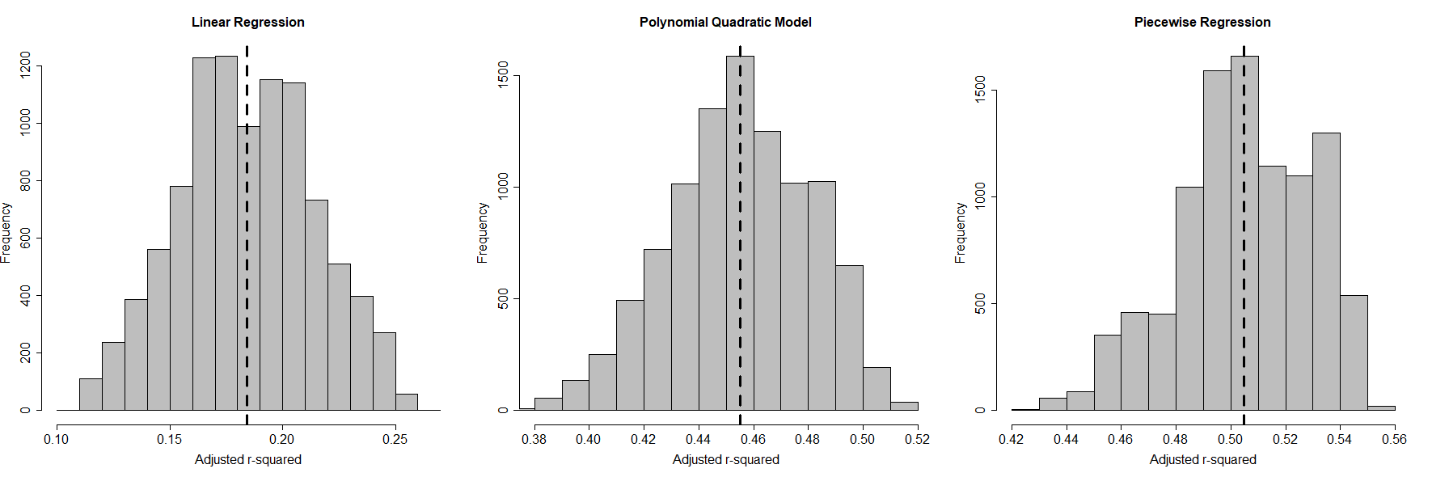


**Figure S2 | Adjusted coefficients of correlation between mean annual precipitation and lineage diversity (standardised effect size of phylogenetic diversity, a metric of evolutionary diversity in communities) using a set of phylogenies that include 68 genera that are present in the genus-by-site matrix but are missing from the original phylogenetic tree.** See Methods for imputation approach. We computed lineage diversity (LD) using the full genus-by-site matrix (920 genera) and a set of 10,000 phylogenetic trees that include the 68 missing genera. These analyses generated 10,000 LD values for each of the 2,025 tree communities, which were used in linear regressions, polynomial quadratic models and piecewise regressions between LD and mean annual precipitation. Histograms represent the frequency of the 10,000 adjusted r-squared values in each method, and dashed line represents their mean adjusted r-squared value.


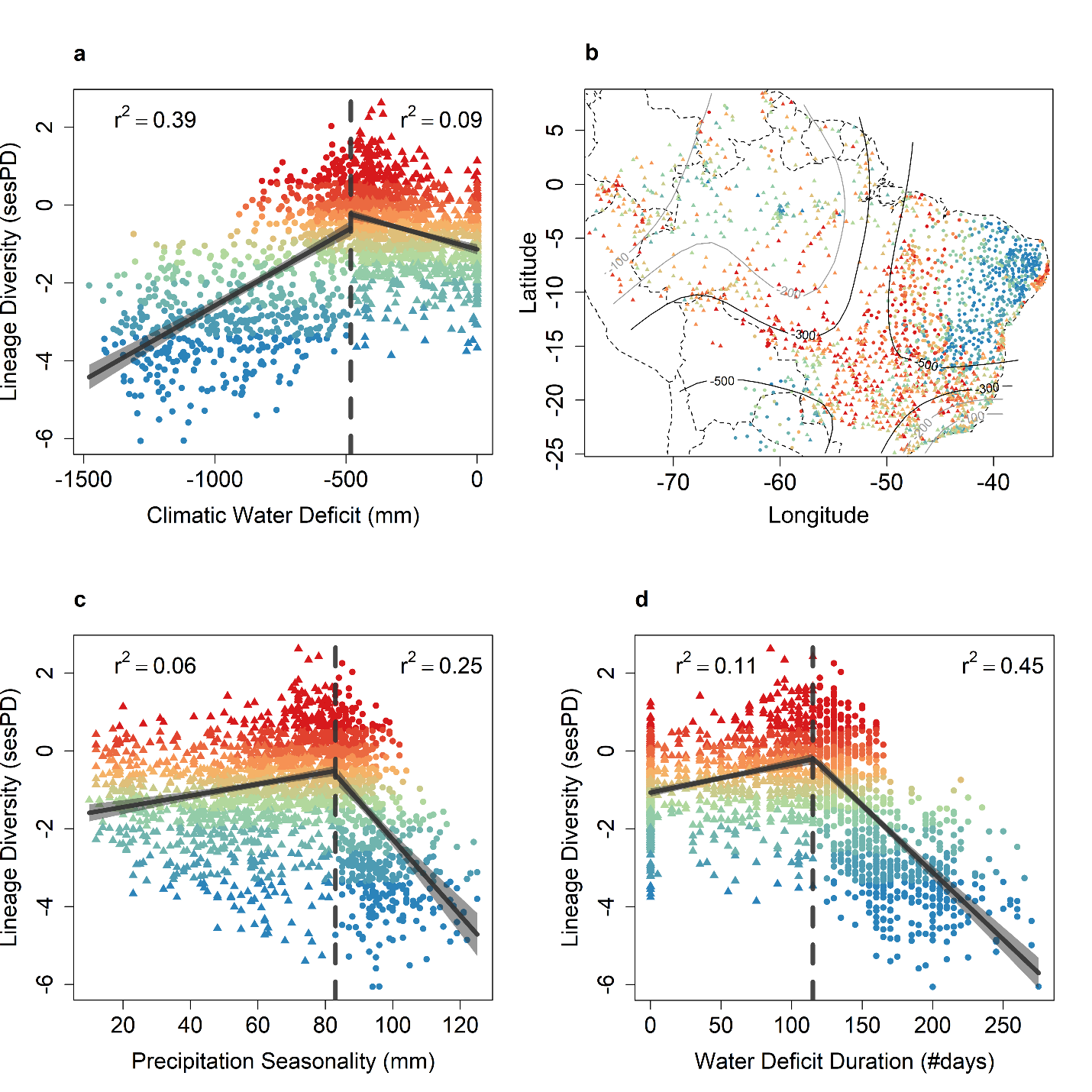


**Figure S3 | Relationship between drought-related variables and lineage diversity (standardised effect size of phylogenetic diversity, a metric of evolutionary diversity in communities) across 2,025 lowland tree communities of tropical South America. a,** Effect of Climatic Water Deficit (mm) on lineage diversity. Break point (-482 mm) was determined by piecewise regression. r^2^ = coefficient of determination from generalized least squares (GLS) models that account for spatial autocorrelation. GLS was calculated for before (y = 0.0038x - 1.2478) and after (y = -0.0019x + 1.1385) the break point. **b,** Geographical variation of lineage diversity and CWD. Colours of the symbols illustrate lineage diversity and are identical to colours in **a** (warmer colours indicate higher values). Circles indicate communities below and triangles above the CWD break point. **c,** Effect of precipitation seasonality on lineage diversity. Break point (83 mm) was determined by piecewise regression. r^2^ = coefficient of determination from generalized least squares (GLS) models that account for spatial autocorrelation. GLS was calculated for before (y = 0.0138x - 1.6976) and after (y = -0.1021x + 7.9406) the break point. **d,** Effect of water deficit duration on lineage diversity. Break point (116 days) was determined by piecewise regression. r^2^ = coefficient of determination from generalized least squares (GLS) models that account for spatial autocorrelation. GLS was calculated for before (y = 0.0074x - 1.067) and after (y = -0.0345x + 3.7772) the break point. Triangles indicate communities below and circles above the break point in **a,** **c** and **d** (CWD -482, 83 mm and 116 days, respectively). Grey areas around the curves represent 99% confidence intervals. Dashed lines represent national borders and contours represent climatic water deficit in **b**.


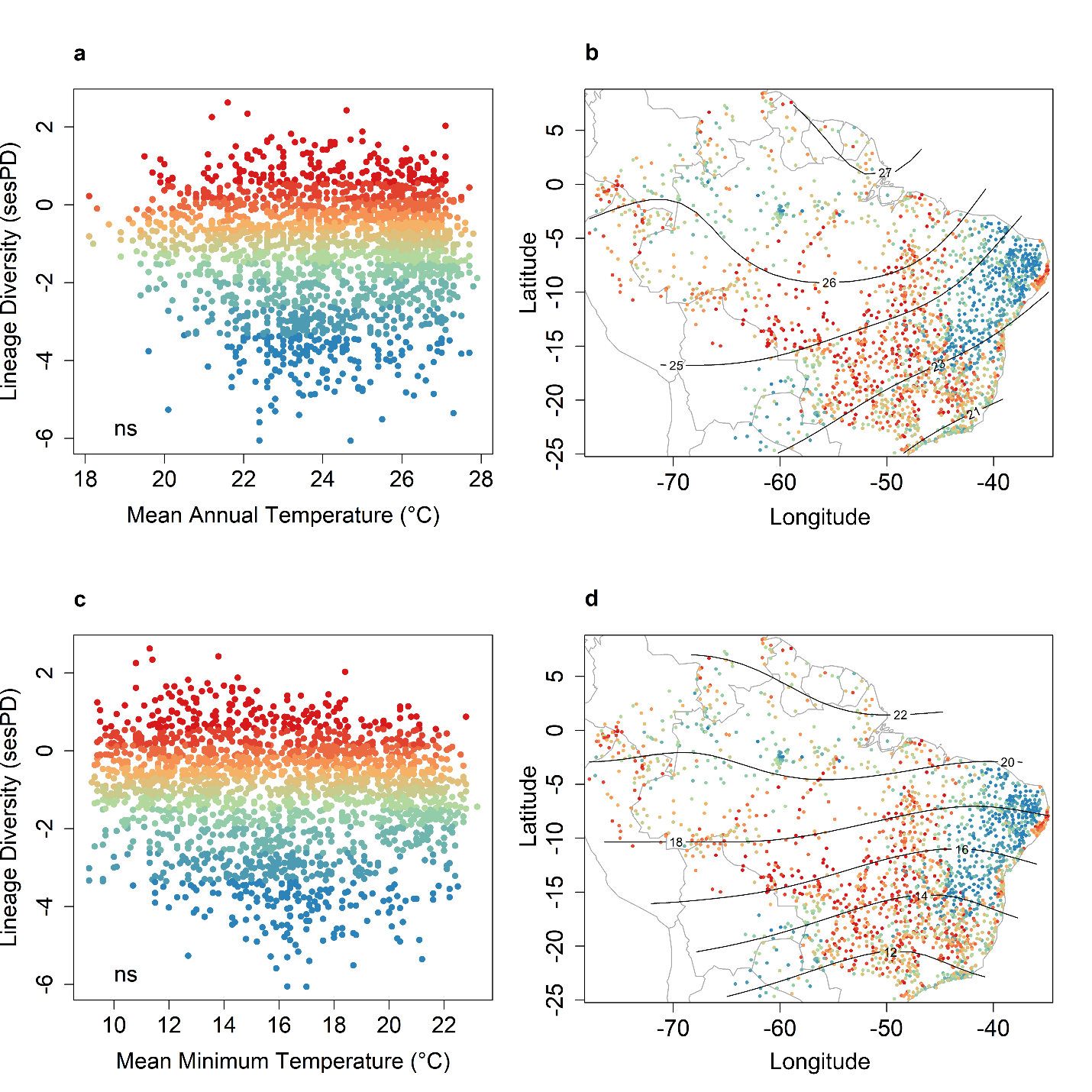


**Figure S4 | Relationship between temperature-related variables and lineage diversity (standardised effect size of phylogenetic diversity) across 2,025 lowland tree communities of tropical South America. a,** Effect of mean annual temperature (MAT) on lineage diversity. **b,** Geographical variation of lineage diversity and MAT. Colours of the symbols illustrate lineage diversity and are identical to colours in **a** (warmer colours indicate higher values). **c,** Effect of mean minimum temperature (TempMin) on lineage diversity. **d,** Geographical variation of lineage diversity and TempMin. Colours of the symbols illustrate lineage diversity and are identical to colours in **c** (warmer colours indicate higher values). ns = non-significant. Grey lines represent national borders. Black contours represent temperature-related variables fitted in geographic space.


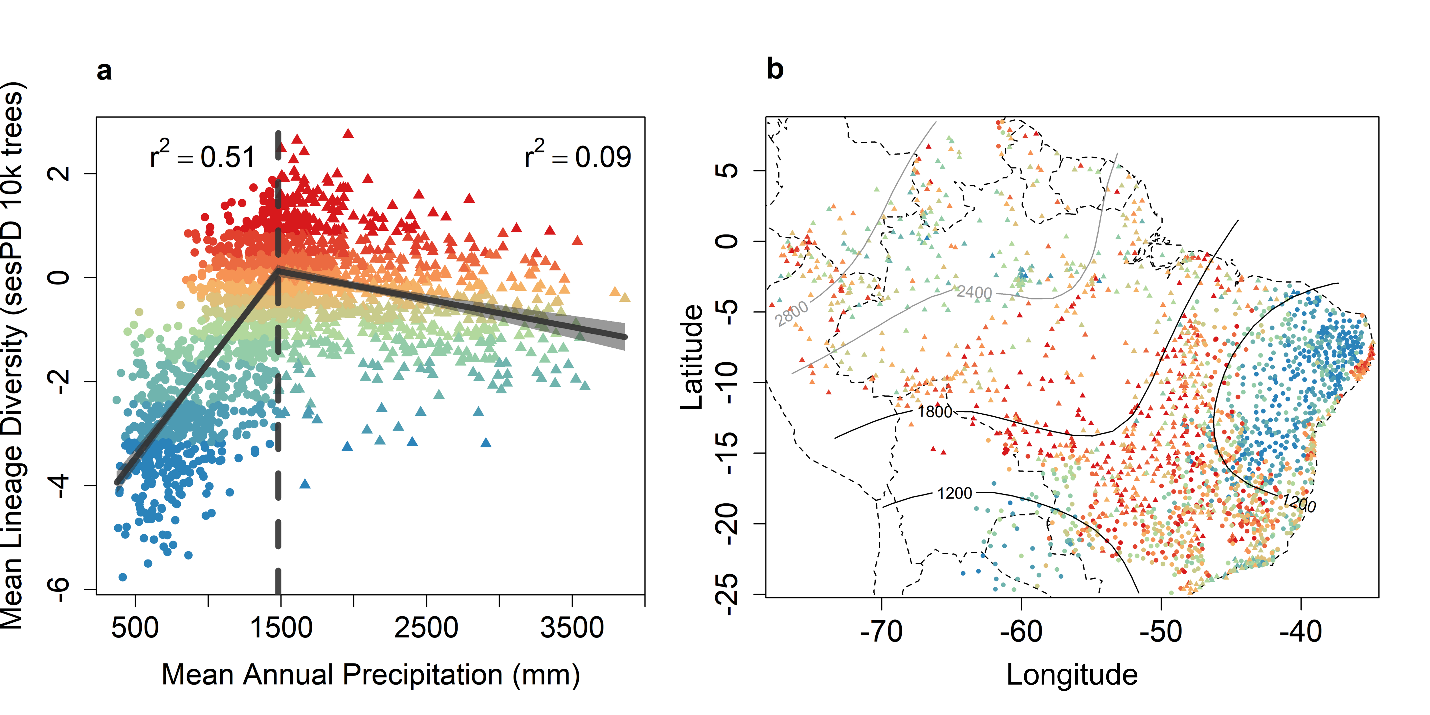


**Figure S5 | Relationship between mean annual precipitation (MAP) and lineage diversity (standardised effect size of phylogenetic diversity) using a set of phylogenies that include 68 genera that are present in the genus-by-site matrix but are missing from the original phylogenetic tree.** See Methods for imputation approach. **a,** Effect of MAP on lineage diversity (LD) across 2,025 lowland tree communities of tropical South America. We computed LD using the full genus-by-site matrix (920 genera) and a set of 10,000 phylogenetic trees that include the 68 missing genera. These analyses generated 10,000 LD values for each of the 2,025 tree communities, which were used to calculate mean values. Break point (1,480 mm) was determined by piecewise regression. r^2^ = coefficient of determination from generalized least squares (GLS) models that account for spatial autocorrelation. GLS was calculated for before (y = 0.0037x - 5.3025) and after (y = -0.0005x + 0.9153) the break point. **b,** Geographical variation of lineage diversity and MAP. Colours of the symbols illustrate lineage diversity and are identical to colours in Fig. 1a (warmer colours indicate higher values). Circles indicate communities below and triangles above the precipitation break point (1,480 mm). Grey areas around the curves in **a** represent 99% confidence intervals. Dashed lines represent national borders and contours represent mean annual precipitation in **b**.


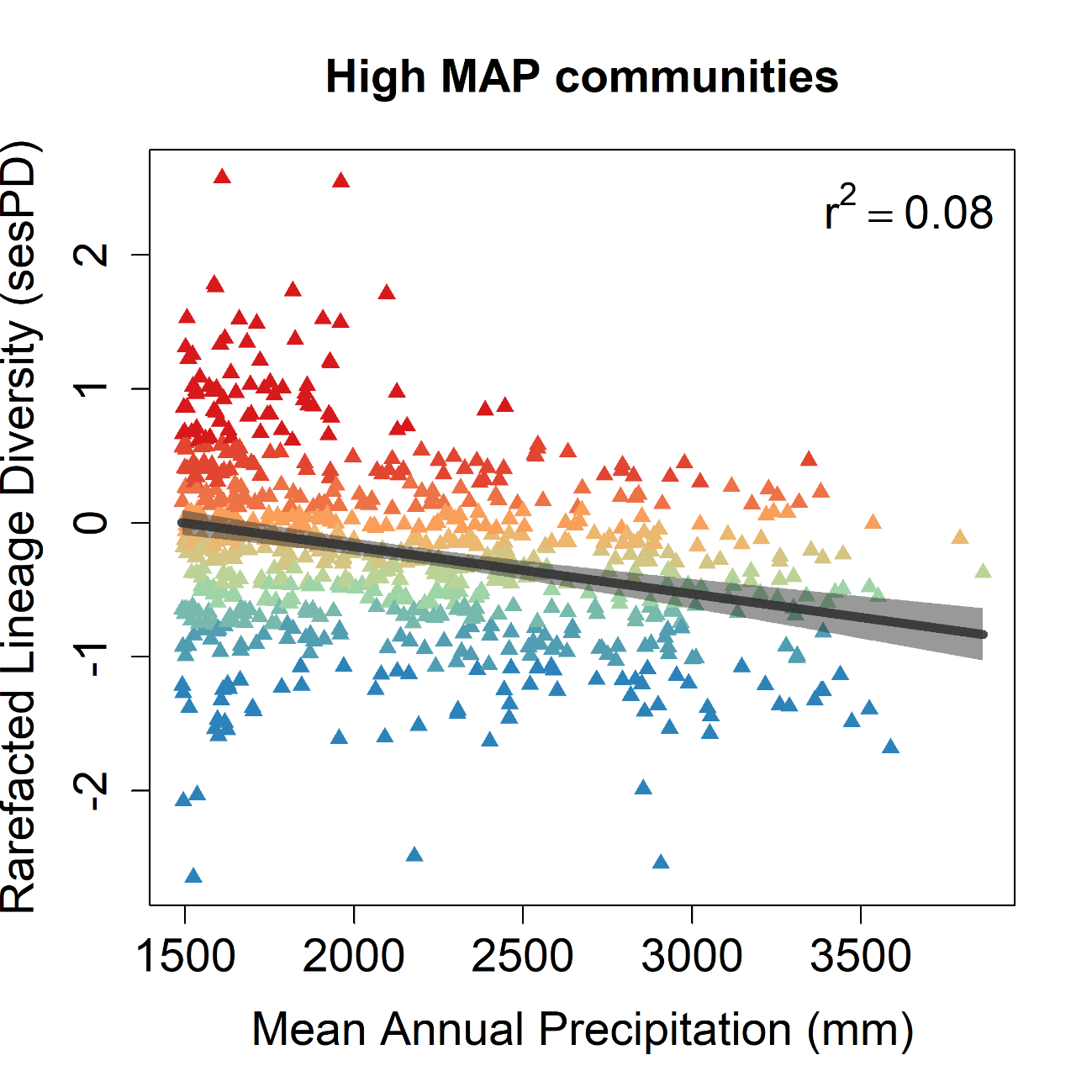


**Figure S6 | Relationship between mean annual precipitation (MAP) and lineage diversity in high MAP communities (MAP** ≥ **1,800mm) using a set of genus-by-site matrices rarefacted to 86 genera.** We computed lineage diversity (LD) using a set of 100 genus-by-site matrices randomly rarefacted to 86 genera, and phylogenetic trees pruned to the genus pool in each matrix. These analyses generated 100 LD values for each of the 519 high MAP communities, which were used to calculate mean values. Grey areas around the curve represent 99% confidence intervals. r^2^ = coefficient of determination from generalized least squares (GLS) models that account for spatial autocorrelation.


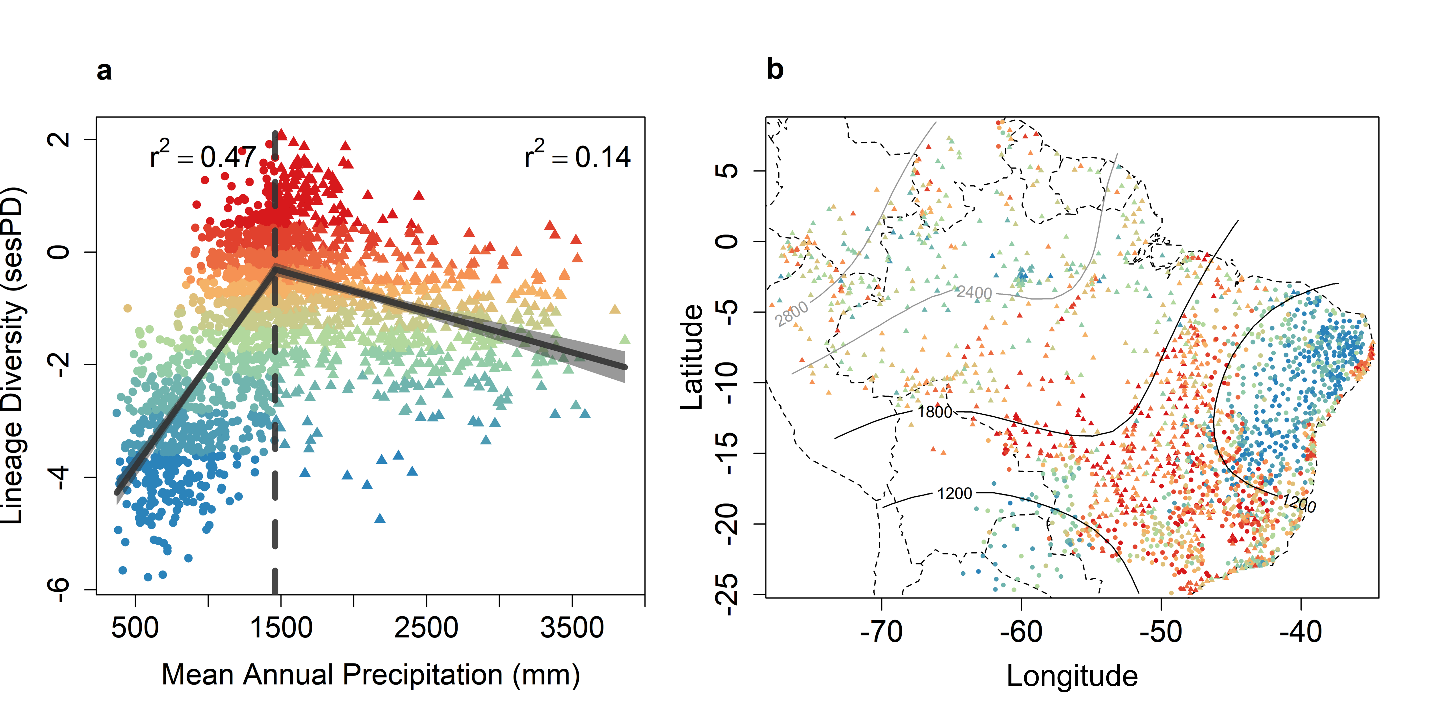


**Figure S7 | Relationship between mean annual precipitation (MAP) and lineage diversity (standardised effect size of phylogenetic diversity) using the most likely phylogeny (from a maximum likelihood analysis) and applying penalised likelihood as an alternative temporal calibration method** (see ‘Phylogenetic tree’ in Supplementary Methods). **a,** Effect of MAP on lineage diversity across 2,025 lowland tree communities of tropical South America. Break point (1,460 mm) was determined by piecewise regression. r^2^ = coefficient of determination from generalized least squares (GLS) models that account for spatial autocorrelation. GLS was calculated for before (y = 0.0036x - 5.6236) and after (y = -0.0007x + 0.7491) the break point. **b,** Geographical variation of lineage diversity and MAP. Colours of the symbols illustrate lineage diversity and are identical to colours in Fig. 1a (warmer colours indicate higher values). Circles indicate communities below and triangles above the precipitation break point (1,460 mm). Grey areas around the curves in **a** represent 99% confidence intervals. Dashed lines represent national borders and contours represent mean annual precipitation in **b**.


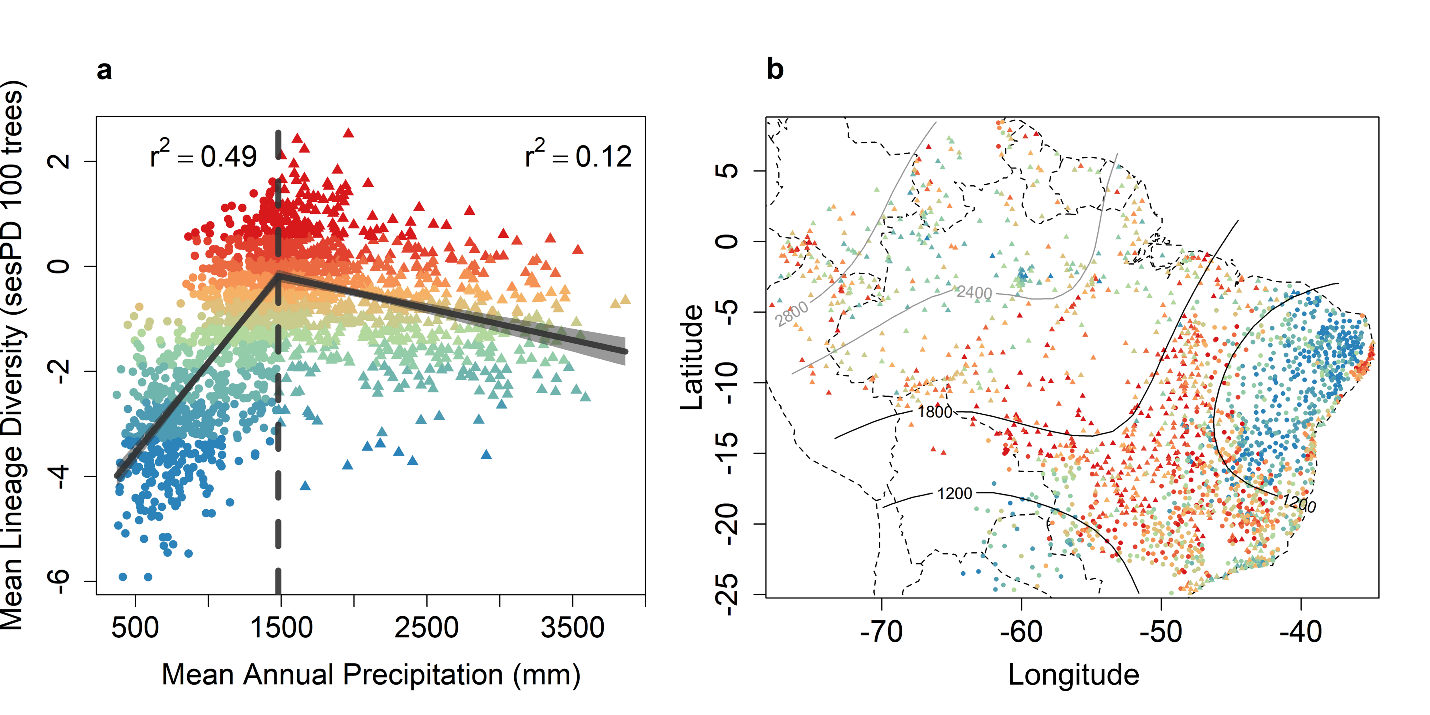


**Figure S8 | Relationship between mean annual precipitation (MAP) and lineage diversity (standardised effect size of phylogenetic diversity) using a set of 100 phylogenies from the posterior distribution**. **a,** Effect of MAP on lineage diversity across 2,025 lowland tree communities of tropical South America. Break point (1,480 mm) was determined by piecewise regression. r^2^ = coefficient of determination from generalized least squares (GLS) models that account for spatial autocorrelation. GLS was calculated for before (y = 0.0034x - 5.2618) and after (y = -0.0006x + 0.7151) the break point. **b,** Geographical variation of lineage diversity and MAP. Colours of the symbols illustrate lineage diversity and are identical to colours in Fig. 1a (warmer colours indicate higher values). Circles indicate communities below and triangles above the precipitation break point (1,480 mm). Grey areas around the curves in **a** represent 99% confidence intervals. Dashed lines represent national borders and contours represent mean annual precipitation in **b**.


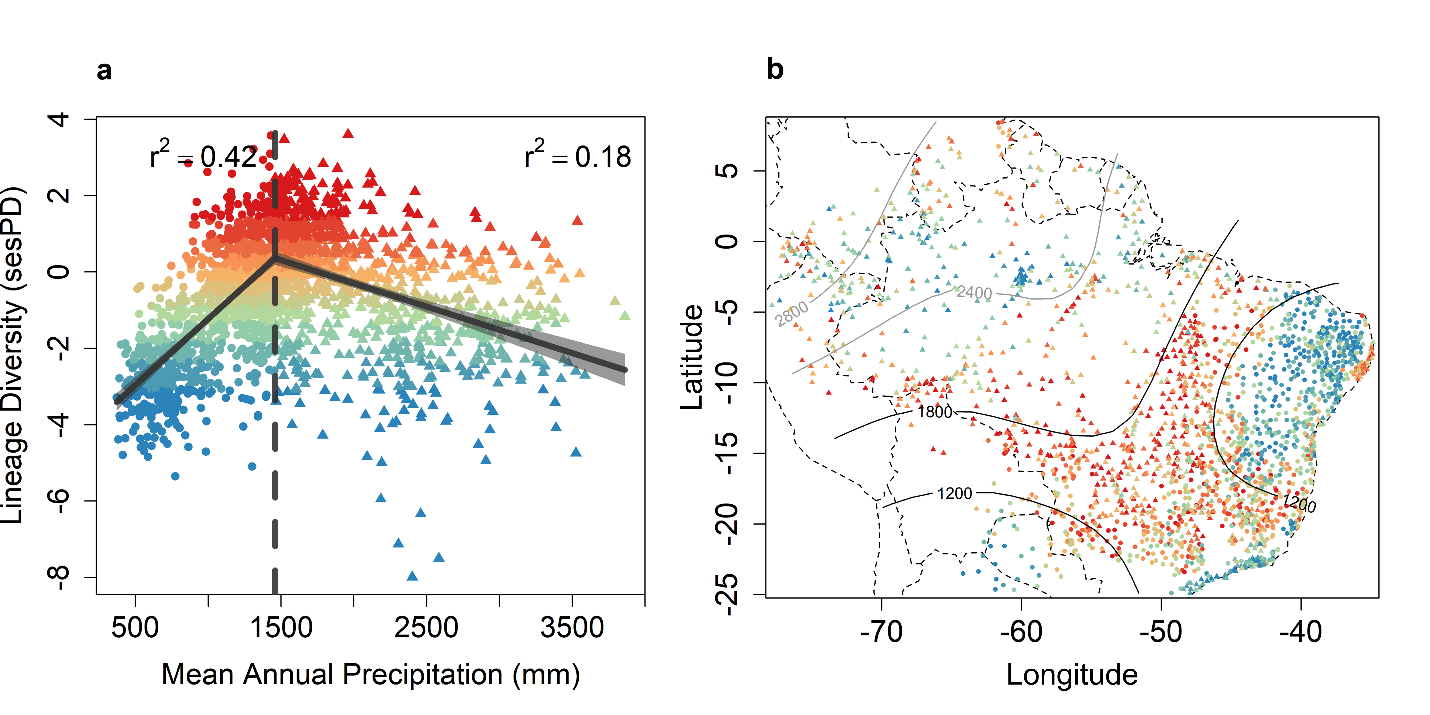


**Figure S9 | Relationship between mean annual precipitation (MAP) and lineage diversity (standardised effect size of phylogenetic diversity) using a phylogeny that includes all 8,174 species in the floristics dataset.** See Supplementary Information for further information regarding the NeoTropTree database. **a,** Effect of MAP on lineage diversity across 2,025 lowland tree communities of tropical South America. Break point (1,460 mm) was determined by piecewise regression. r^2^ = coefficient of determination from generalized least squares (GLS) models that account for spatial autocorrelation. GLS was calculated for before (y = 0.0034x - 4.6767) and after (y = -0.0012x + 2.1599) the break point. **b,** Geographical variation of lineage diversity and MAP. Colours of the symbols illustrate lineage diversity and are identical to colours in Fig. 1a (warmer colours indicate higher values). Circles indicate communities below and triangles above the precipitation break point (1,460 mm). Grey areas around the curves in **a** represent 99% confidence intervals. Dashed lines represent national borders and contours represent mean annual precipitation in **b**.

**References**

54. Oliveira-Filho, A. T. Classification of vegetation physiognomies of tropical and subtropical cis-Andean South America: proposal of a practical and flexible new system or an additional injection of chaos? *Rodriguesia* **60**, 237-258 (2009).

55. Zak M. R., Cabido, M. & Hodgson J. G. Do subtropical seasonal forests in the Gran Chaco, Argentina, have a future? Biol. Conserv. **120**, 589-598 (2004).

56. Hopkins, M. J. G. Modelling the known and unknown plant biodiversity of the Amazon Basin. *J. Biogeogr.* **34**, 1400-1411 (2007).

57. Ministério do Meio Ambiente (Ministry of Environment, Brazil) *Mapa de cobertura vegetal dos biomas brasileiros*. Ministério do Meio Ambiente, Brasília (2007). Available at: https://www.mma.gov.br/informma/item/3769-mma-lanca-mapas-de-cobertura-vegetal-nativa-dos-biomas-brasileiros

58. Fiaschi, P. & Pirani, J. R. Review of plant biogeographic studies in Brazil. *J. Syst. Evol.* **47**, 477-496 (2007).

59. Forzza, R. C. *et al.* New Brazilian floristic list highlights conservation challenges. *BioScience* **62**, 39-45 (2012).

60. Prado, D. E. & Gibbs, P. E. Patterns of species distribution in the dry seasonal forests of South America. *Ann. Mo. Bot. Gard.* **80**, 902–927 (1993).

61. Ratter, J. A., Askew, G. P., Montgomery, R. & Gifford, D. R. Observations on forests of some mesotrophic soils in Central Brazil. *Revista Brasileira de Botânica* **1**, 47-58 (1978).

62. Pennington, R. T.; Prado, D. E. & Pendry, C. A. Neotropical seasonally dry forests and Quaternary vegetation changes. *J. Biogeogr.* **27**, 261–273 (2000).

63. Oliveira-Filho, A. T., Jarenkow, J. A. & Rodal, M. J. N. in *Neotropical savannas and dry forests: plant diversity, biogeography and conservation* (eds Pennington R. T., Ratter J. A. & Lewis G. P.) 151-184 (CRC Press, Boca Raton, 2006).

64. Neves, D. M., Dexter, K. G., Pennington, R. T., Bueno, M. L. & Oliveira-Filho, A. T. Environmental and historical controls of floristic composition across the South American Dry Diagonal. *J. Biogeogr.* **42**, 1566-1576 (2015).

65. Silva de Miranda, P. L. *et al.* Using tree species inventories to map biomes and assess their climatic overlaps in lowland tropical South America. *Global Ecol. Biogeogr.* **27**, 899– 912 (2018).

66. Hijmans, R. J., Cameron, S. E., Parra, J. L., Jones, P. G. & Jarvis, A. Very high resolution interpolated climate surfaces for global land areas. *Int.* *J. Climatol.* **25**, 1965–1978 (2005).

67. Walter, H. *Vegetation of the earth and ecological systems of the geo-biosphere*, 3rd edn. Springer-Verlag, Berlin (1985).

68. Chave, J. *et al.* Improved allometric models to estimate the aboveground biomass of tropical trees. *Glob. Change Biol.* **20**, 3177-3190 (2014).

69. Dexter, K. G. & Chave, J. Evolutionary patterns of range size, abundance and species richness in Amazonian trees. *PeerJ*, 2043v1 (2016).

70. CBOL Plant Working Group. A DNA barcode for land plants. *Proc. Natl. Acad. Sci. USA* **106**, 12794-12797 (2009).

71. Kress, W. J. & Erickson D. L. DNA barcodes: methods and protocols. *Methods Mol. Biol.* **858**, 3-8 (2012).

72. Kress, W. J., Lopez, I. C. & Erickson, D. L. Generating plant DNA barcodes for trees in long-term forest dynamics plots. *Methods Mol. Biol.* **858**, 441-458 (2012).

73. Gonzalez, M. A. *et al.* Identification of Amazonian trees with DNA barcodes. *PLoS One* **4**, e7483 (2009).

74. Katoh, K., Misawa, K., Kuma, K. & Miyata, T. MAFFT: a novel method for rapid multiple sequence alignment based on fast Fourier transform. *Nucleic Acids Res.* **30**, 3059-3066 (2002).

75. The Angiosperm Phylogeny Group. An update of the Angiosperm Phylogeny Group classification for the orders and families of flowering plants: APG IV. *Bot. J. Linn. Soc.* **181**, 1-20 (2016).

76. Paradis, E., Claude, J. & Strimmer, K. APE: analyses of phylogenetics and evolution in R language. *Bioinformatics* **20**, 289-290 (2004).

77. Dummond, A. J. & Rambaut, A. BEAST Bayesian evolutionary analysis by sampling trees. *BMC Evo. Biol.* **7**, 214 (2007).

78. Stadler, T. On incomplete sampling under birth-death models and connections to the sampling-based coalescent. *J. Theor. Biol.* **261**, 58-66 (2009).

79. Baker, T. R. *et al.* Fast demographic traits promote high diversification rates of Amazonian trees. *Ecol. Lett.* **17**, 527-536 (2014).

80. Magallon, S., Gomez-Acevedo, S., Sanchez-Reyes, L. L. & Hernandez-Hernandez T. A metacalibrated time-tree documents the early rise of flowering plant phylogenetic diversity. *New Phytol.* **207**, 437-453 (2015).

81. Stadler, T. TreeSim: Simulating Phylogenetic Trees. R package version 2.3 (2017). Available at: https://CRAN.R-project.org/package=TreeSim
